# Early Pregnancy Diagnosis in Sows: A Comparative Evaluation of Ultrasonographic and Progesterone-Based Methods

**DOI:** 10.3390/life16050854

**Published:** 2026-05-21

**Authors:** Georgi Garbev, Stanimir Dimitrov

**Affiliations:** Department of Fundamental Sciences in Animal Husbandry, Section of Reproduction, Faculty of Agriculture, Trakia University, 6000 Stara Zagora, Bulgaria; stanimir.dimitrov@trakia-uni.bg

**Keywords:** reproduction in sows, early pregnancy diagnosis, ultrasonography, progesterone assay, reproductive management

## Abstract

Early pregnancy diagnosis is a key component of reproductive management in swine production systems. Accurate identification of pregnant and non-pregnant sows within the first 30 days after insemination allows timely reproductive decisions and reduces non-productive days. The present systematic review evaluates the diagnostic efficiency of ultrasonographic and progesterone-based methods used for early detection of pregnancy in sows. A structured literature search was conducted in accordance with the PRISMA Statement guidelines, using major scientific databases. Studies evaluating pregnancy diagnosis in sows within the first 30 days after insemination were included. Diagnostic approaches were analyzed with respect to methodological design, timing of examination, biological sample matrix, and reported indicators of diagnostic accuracy. Ultrasonographic techniques have evolved from early acoustic detection in A-mode to real-time imaging in B-mode and more recently algorithm-assisted interpretation of ultrasound images. Real-time ultrasonography allows direct visualization of gestational structures; in one study, diagnostic accuracy above 95% was reported after approximately 23–24 days of pregnancy under optimal examination conditions. Progesterone-based analyses evaluate luteal endocrine activity and are particularly useful for early identification of non-pregnant animals after luteolysis. The diagnostic efficiency of hormonal assays depends strongly on the timing of sampling and the biological matrix used for analysis, including plasma, serum, dried blood spots, saliva, or feces. The comparative analysis shows that ultrasonography provides morphological confirmation of pregnancy, whereas progesterone analyses serve mainly as functional indicators of luteal activity. These methods play complementary roles in reproductive management. Ultrasonography remains the most reliable method for confirming pregnancy, while progesterone-based analyses are valuable tools for early reproductive screening and identification of non-pregnant sows.

## 1. Introduction

Early pregnancy diagnosis in sows is an essential element of reproductive management in industrial swine production. Timely identification of non-pregnancy allows optimization of the interval between inseminations and reduction of non-productive days. Its practical importance is greatest within the first 30 days after insemination, a period that includes maternal recognition of pregnancy and early embryonic development.

Ultrasonographic diagnosis was introduced in the mid-1970s through amplitude–depth analysis (A-mode), in which reflections of the ultrasound signal from the presence of fluid in the uterine horns are recorded [1]. The development of real-time ultrasonography (B-mode) allows direct visualization of gestational vesicles and embryonic structures [2,3]. Subsequent studies evaluated diagnostic accuracy under field conditions and analyzed factors associated with false-positive diagnoses [4,5]. Recent developments include the use of image processing algorithms and machine learning-based models for automated interpretation of ultrasound data [6,7].

In parallel with imaging methods, hormonal approaches based on the determination of progesterone as an indicator of persistent luteal function have been developed. Enzyme and radioimmunoassay methods for progesterone determination in plasma were validated as early as the 1980s [8,9]. Later, alternative biological samples, including saliva and feces, were investigated [10,11], as well as field enzyme-linked immunosorbent assay (ELISA) systems [12]. In addition, estrone sulfate has been considered as a marker associated with early embryonic development [13,14].

Despite the long-term development of these diagnostic technologies, published studies use different time intervals of examination, different analytical thresholds, and different criteria for confirmation of pregnancy. These differences limit direct comparison of diagnostic accuracy during early gestation.

This systematic review aimed:

(1) to evaluate the diagnostic accuracy of ultrasonographic methods (A-mode, real-time ultrasonography, and image processing algorithms) for early pregnancy diagnosis in sows (≤30 days after insemination);
(2) to evaluate the diagnostic accuracy of progesterone-based methods, including different biological samples and analytical techniques;
(3) to perform a comparative analysis of sensitivity, specificity, time window of application, and the frequency of false-positive and false-negative results.

## 2. Materials and Methods

### 2.1. Literature Search Strategy

This systematic review was conducted in accordance with the PRISMA guidelines.

A comprehensive literature search was performed in Web of Science, Scopus, and PubMed to identify studies on early pregnancy diagnosis in sows. The search covered publications from January 1975 to March 2025.

The search strategy was developed using combinations of controlled vocabulary and free-text terms related to the target species, diagnostic methods, and early pregnancy detection. The search terms were structured into three main components:

Species (“sow”, “pig”), condition *(*“*pregnancy diagnosis*”, “*early pregnancy*”), and diagnostic approach (“*ultrasonography*”, “*ultrasound*”, “*progesterone*”, “*ELISA*”, “*gestagens*”).

A representative search strategy was:

(“*sow*” OR “*pig*”) AND (“*pregnancy diagnosis*” OR “*early pregnancy”*) AND (“*ultrasonography*” OR “*ultrasound*” OR “*progesterone*” OR “*ELISA*” OR “*gestagens*”).

The search strategy was adapted to the syntax and indexing of each database as follows:

#### 2.1.1. Web of Science

(TS = (“sow” OR “pig”) AND

TS = (“pregnancy diagnosis” OR “early pregnancy”) AND

TS = (“ultrasonography” OR “ultrasound” OR “progesterone” OR “ELISA” OR “gestagens”));

#### 2.1.2. Scopus

(TITLE-ABS-KEY(“sow” OR “pig”) AND

TITLE-ABS-KEY(“pregnancy diagnosis” OR “early pregnancy”) AND

TITLE-ABS-KEY(“ultrasonography” OR “ultrasound” OR “progesterone” OR “ELISA” OR “gestagens”));

#### 2.1.3. PubMed

((“sow”[Title/Abstract] OR “pig”[Title/Abstract]) AND

(“pregnancy diagnosis”[Title/Abstract] OR “early pregnancy”[Title/Abstract]) AND

(“ultrasonography”[Title/Abstract] OR “ultrasound”[Title/Abstract] OR “progesterone”[Title/Abstract] OR “ELISA”[Title/Abstract] OR “gestagens”[Title/Abstract])).

This search structure was applied across all databases with minor adaptations where required. Only articles published in English were included. No additional filters were applied. In addition to database searching, the reference lists of relevant studies were manually screened to identify additional eligible publications. CAB Abstracts was considered as an additional potentially relevant database; however, it was not included in the final search strategy because Web of Science, Scopus, PubMed, and citation screening were considered to provide sufficient coverage of the peer-reviewed literature relevant to early pregnancy diagnosis in sows.

### 2.2. Eligibility Criteria

Following the literature search, studies were selected for inclusion based on predefined eligibility criteria.

#### 2.2.1. Inclusion Criteria

Studies were included if they met the following criteria:

- original research articles published in peer-reviewed journals
- studies involving pregnancy in sows or gilts
- studies evaluating ultrasonographic methods or progesterone-based diagnostic approaches
- studies focused on early pregnancy diagnosis (≤30 days after insemination)
- studies providing a methodological description of the diagnostic approach

#### 2.2.2. Exclusion Criteria

Studies were excluded if they met any of the following criteria:

- review articles
- publications without original experimental data
- studies not addressing pregnancy diagnosis in sows
- studies focused on late pregnancy diagnosis (>30 days after insemination)
- studies lacking sufficient methodological information on the diagnostic method used

### 2.3. Study Selection

The study selection process was conducted in accordance with the PRISMA guidelines and followed a structured, multi-stage approach.

A total of 198 records were identified, including 186 records from database searching and 12 additional records identified through citation screening. After removal of duplicate records (n = 50), 148 records remained.

Titles and abstracts were screened independently by two reviewers to assess relevance. Following this stage, 52 studies were selected for full-text assessment. Full-text articles were then evaluated against the predefined eligibility criteria. A total of 30 studies met the inclusion criteria and were included in the systematic review. Of the 22 excluded studies, 7 were review articles, 8 lacked sufficient methodological data, and 7 were not focused on early pregnancy diagnosis. Any discrepancies during the screening and selection process were resolved through discussion. The studies included after the selection process were then subjected to data extraction. A detailed description of the study selection process, including reasons for exclusion, is presented in Table 1.

### 2.4. Data Extraction

Data extraction was performed independently by two reviewers using a standardized and pre-tested data extraction form developed for this systematic review.

The extracted information included:

- diagnostic method used
- type of equipment or analytical test used
- type of biological sample
- timing of diagnostic examination after insemination
- diagnostic threshold used (cut-off values in hormonal assays)
- reported indicators of diagnostic accuracy

Discrepancies between reviewers were resolved through discussion and consensus after rechecking the original publications.

When relevant data were missing or not clearly reported, they were recorded as not reported and were not used for direct comparison of diagnostic performance, but were considered in the qualitative synthesis when appropriate.

The authors of the original studies were not contacted for missing data.

The extracted data were used to support the structured comparison of ultrasonographic and progesterone-based methods for early pregnancy diagnosis in sows.

### 2.5. Risk of Bias Assessment

The methodological quality and risk of bias of the included diagnostic accuracy studies were assessed using the QUADAS-2 tool.

The assessment was performed across four domains: patient selection, index test, reference standard, and flow and timing. Each domain was classified as low, high, or unclear risk of bias based on the reported methodology of each study. Only studies evaluating diagnostic accuracy of pregnancy detection methods were included in the risk of bias asessment. Studies focused on artificial intelligence model development, biomarker discovery, or non-diagnostic outcomes were not subjected to formal QUADAS-2 assessment.

The results of the risk of bias assessment are presented in Supplementary Table S1.

The findings were considered during the interpretation of diagnostic performance, particularly when comparing studies with different reference standards and incomplete reporting.

### 2.6. Data Synthesis

Due to substantial heterogeneity among the included studies in terms of diagnostic protocols, timing of examination, biological samples, reference standards, and reported outcome measures, a quantitative meta-analysis was not performed.

The synthesis of the evidence was conducted using a structured narrative approach. Studies were systematically categorized according to diagnostic method (ultrasonography versus progesterone-based assays), timing of examination after insemination, and type of biological sample. Within each category, studies were compared with respect to reported diagnostic performance indicators, methodological characteristics, and potential sources of diagnostic error. Particular attention was given to differences in study design and to the variability in reference standards used for pregnancy confirmation (e.g., ultrasonographic diagnosis versus farrowing outcome), which may influence reported specificity and sensitivity.

The heterogeneity observed across studies was considered substantial from clinical, methodological, and analytical perspectives, which precluded meaningful quantitative pooling of results. No subgroup meta-analysis or partial pooling was attempted due to the lack of sufficiently comparable and consistently reported data.

The results of the risk of bias assessment were taken into account during the synthesis and interpretation of findings.

**Figure 1:**
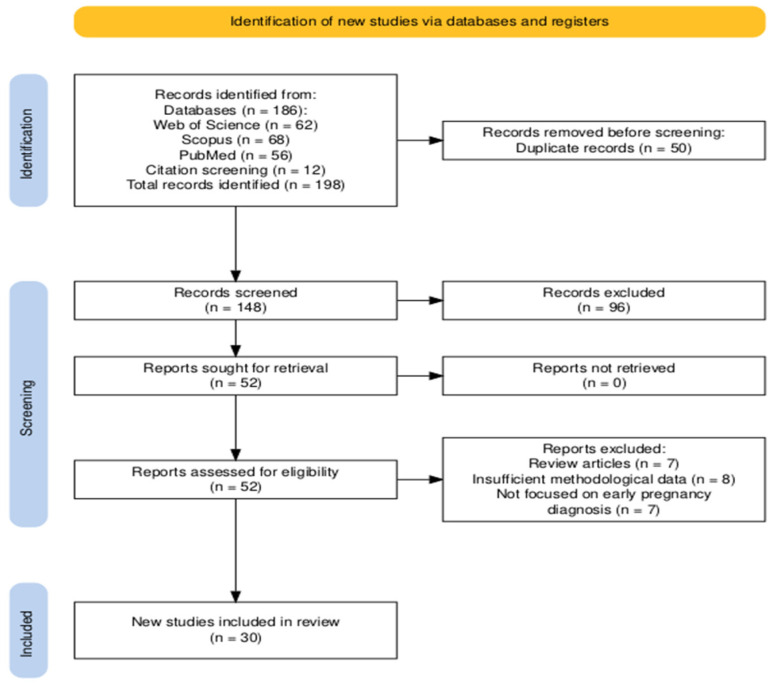
PRISMA 2020 flow diagram showing the identification, screening, eligibility assessment, and inclusion of studies in the systematic review.

The PRISMA flow diagram summarizing the study selection process is presented in Figure 1.

## 3. Results

### 3.1. Characteristics of Included Studies

A total of 30 scientific publications investigating methods for early pregnancy diagnosis in sows were included in the systematic review (Table 2). The studies can be grouped into three main categories according to the diagnostic principle:

(1) ultrasonographic methods,
(2) progesterone-based hormonal methods, and
(3) alternative endocrine or biomarker approaches, including estrogen metabolites and novel biomarkers.

This categorization was used as the basis for the structured comparison of diagnostic approaches in the subsequent analysis.

Earlier studies (1970s and 1980s) are focused on the development of ultrasonographic diagnosis and hormonal tests. More recent publications include advanced imaging technologies and image processing algorithms, as well as novel biomarkers for early pregnancy. Across the included studies, substantial variability was observed in study design, timing of examination, biological samples, reference standards used to confirm pregnancy, and reporting of diagnostic performance. This variability was considered when comparing the diagnostic value and practical applicability of the different approaches.

Studies reporting NR for diagnostic performance metrics were retained in the review because they provided relevant methodological information on diagnostic principles, timing of application, biological samples, or reference standards. However, these studies were not used for direct comparison of sensitivity, specificity, or accuracy.

Studies involving artificial intelligence or machine learning approaches were interpreted separately from conventional ultrasonographic methods because most of them were based on image datasets or algorithm development rather than routine field validation. Therefore, their diagnostic performance was considered preliminary and not directly comparable with results obtained using conventional on-farm ultrasound examination.

Differences in reference standards, including pregnancy status at scanning, ultrasonographic confirmation, and farrowing outcome, were considered an important source of variability. These differences may affect the interpretation of specificity and overall diagnostic accuracy across studies.

### 3.2. Ultrasonographic Methods for Early Pregnancy Diagnosis in Sows

#### 3.2.1. Evolution of Ultrasonographic Techniques and Diagnostic Principle

Ultrasonographic diagnosis of early pregnancy in sows has evolved from indirect acoustic techniques to direct imaging visualization and finally to algorithmic interpretation of digital images. The earliest publications used A-mode ultrasound, in which the diagnostic conclusion is based on detection of an echo signal associated with the presence of fluid in the uterine horns, without direct visualization of gestational structures. Lindahl et al. [1] demonstrated that amplitude–depth analysis allows early detection of pregnancy in sows, but the diagnostic principle remains indirect, as it detects acoustic reflection from fluid rather than embryonic structures. A similar principle is applied in pulse-mode ultrasound, described by Holtz [15], where the acoustic signal is interpreted without morphological visualization. Pyörälä [16] compared A-mode ultrasound with palpation and showed that ultrasound is more reliable than clinical examination, but the limitations of the indirect signal remain.

The transition to real-time B-mode ultrasonography substantially changes the diagnostic principle. Inaba et al. [2] introduced linear electronic scanning, which produces a real image of uterine structures. The available record indicates a frequency of 3.5 MHz, which is important as it demonstrates the early use of a lower-frequency transducer to achieve better tissue penetration in transabdominal examination. Taverne et al. [3] compared linear-array real-time scanning with amplitude–depth analysis under field conditions and show that visualization of the uterine horns and gestational structures leads to higher diagnostic reliability. In B-mode ultrasonography, the diagnosis is not based on an indirect acoustic effect but on morphological evaluation of the image, which represents a methodologically different and stronger approach. Later studies further develop this model by introducing more precise criteria for ultrasonographic evaluation. De Rensis et al. [17] used not only the presence of gestational vesicles but also uterine echotexture and embryonic development as diagnostic indicators in the period 15–25 days after insemination. This extends ultrasonographic diagnosis beyond the binary distinction “pregnant/non-pregnant” and directs it toward evaluation of the biological validity of early pregnancy.

The current stage of development includes automated processing of ultrasound images. Kousenidis et al. [6] used a machine learning model to predict litter size based on ultrasonographic data, which is not a direct test for pregnancy but demonstrates that ultrasound information can be used quantitatively. Chae et al. [19] developed an algorithm for automated pregnancy diagnosis based on ultrasound images. Kim et al. [7] used a modified YOLOv7 model for detection of the gestational sac, and Kim et al. [20] applied deep learning analysis to low-frequency ultrasound images. These studies extend the interpretation of ultrasound data from operator-based visual assessment to automated image analysis. However, they do not change the biological basis of ultrasonographic pregnancy diagnosis, which remains dependent on the detection or visualization of pregnancy-associated uterine and embryonic structures.

Critical analysis

The development from A-mode to real-time B-mode and subsequently to AI-based approaches does not represent only technological modernization but a change in the diagnostic principle. A-mode and pulse-mode rely on indirect detection of fluid and are therefore more susceptible to non-specific echo signals. B-mode allows direct visualization and is therefore methodologically more reliable. Algorithmic models improve standardization of interpretation but do not change the fact that diagnostic quality continues to depend on the timing of examination, image quality, and clinical context. Because most AI-based studies have been evaluated using limited image datasets rather than large-scale field validation, their results should be interpreted as preliminary and should not be directly equated with routine clinical diagnosis at the animal level.

#### 3.2.2. Diagnostic Performance Reported in the Literature

The reported results clearly show that the diagnostic accuracy of ultrasonographic methods in sows is strongly dependent on gestational age. In A-mode and pulse-mode, publications demonstrate applicability in early pregnancy, but in most available records, standardized sensitivity and specificity are lacking, which makes direct quantitative comparison difficult. The study by Pyörälä [16] is one of the few early reports in which specific diagnostic indicators are available: for A-mode ultrasound, an overall accuracy of 96.8% and sensitivity of 98.9% are reported. These values indicate a high ability to identify pregnant animals, but without direct data on specificity, the interpretation remains incomplete.

In real-time ultrasonography, quantitative information is more consistent. The most detailed temporal analysis is presented by Maes et al. [5], who examine sows transabdominally between day 16 and day 25 after insemination, using two types of probes: a linear transducer (5 MHz) and a sector transducer (3.5 MHz). The lower-frequency 3.5 MHz sector probe provides greater tissue penetration, which is particularly relevant in larger sows, whereas the 5 MHz linear probe offers higher image resolution and may improve visualization of early gestational vesicles. The first ultrasonographic detection of pregnancy is possible around day 18. Sensitivity, specificity, and overall accuracy above 95% are achieved from day 24 with the linear probe and from day 23 with the sector probe. This is one of the most important observations in the literature, as it shows that even within the same diagnostic principle, the type of probe and its frequency can influence the threshold for reliable diagnosis. Szenci et al. [4] analyze diagnosis between day 26 and day 30 after insemination and report a sensitivity of 99% and specificity of 73.1% when farrowing is used as the reference standard. Gokuldas et al. [18], in a field study under intensive management conditions, report a sensitivity of 84.21% and specificity of 75% for diagnosis before day 30. These results are lower than the highest values reported under controlled conditions but are particularly important because they reflect real farm conditions. For algorithm-based methods, Chae et al. [19] report a sensitivity of 0.9876 and specificity of 0.9982 in image analysis, indicating very high internal model accuracy. Kim et al. [7] report a mean average precision of 89.8% for detection of the gestational sac using YOLOv7-E6E. Kim et al. [20] report an AUC of approximately 0.86 in deep learning analysis of low-frequency ultrasound images. These indicators demonstrate the potential of automated image analysis, but they were obtained from experimental image datasets and should not be interpreted as equivalent to animal-level diagnostic accuracy under routine farm conditions.

Critical analysis

The reported indicators of ultrasonographic diagnosis are not fully comparable across studies. This limited comparability is partly explained by differences in diagnostic objectives: some publications assess the presence of pregnancy at the animal level, others evaluate algorithm performance on images, and others use the final reproductive outcome as a reference standard. This means that a sensitivity of 99% in a clinical study and a sensitivity of 0.9876 in an image-based algorithm are not equivalent indicators. In addition, high-performance values under experimental conditions do not guarantee identical effectiveness in real farm use. Table 3 presents the diagnostic performance of ultrasonographic approaches.

#### 3.2.3. Methodological Heterogeneity Among Studies

The included ultrasonographic studies differ substantially in study design and diagnostic protocols, which limits direct comparison of reported diagnostic accuracy. This heterogeneity is related to several dimensions, including day of examination, type and frequency of the probe, reference standard, population, study setting, and unit of analysis.

Gestational age at examination varies considerably. De Rensis et al. [17] analyzed the period 15–25 days after insemination, Maes et al. [5] used a daily protocol between days 16 and 25, whereas Szenci et al. [4] and Gokuldas et al. [18] evaluated diagnosis in a later window, close to days 26–30. This is critical, because the size of the gestational vesicle and the amount of intrauterine fluid change rapidly during this period, which directly affects the probability of detection. Therefore, studies conducted on day 18 and day 28 cannot be interpreted as being performed under equivalent diagnostic conditions. Probe type and frequency also contribute to methodological variability. Maes et al. [5] showed that a sector transducer (3.5 MHz) and a linear transducer (5 MHz) did not provide identical temporal thresholds for achieving high diagnostic accuracy. This demonstrates that probe frequency and geometry have a real impact on clinical outcome and are not merely technical details.

The reference standard varies across publications. Some studies used farrowing as the endpoint, whereas others relied on repeated ultrasonographic confirmation or pregnancy status at the time of examination. When farrowing is used as the reference standard, diagnostic accuracy reflects not only the ability to detect pregnancy early but also subsequent embryonic or fetal losses until term. As a result, a true pregnancy detected at the time of scanning may be classified as false positive if the animal does not farrow, which may reduce apparent specificity compared with designs in which pregnancy is assessed at the time of testing.

The study populations and settings are also heterogeneous. Some studies were field-based and included animals under routine production conditions, whereas others were experimental or image-based. Field studies reflect the effects of management, animal condition, working conditions, and operator experience. This increases external validity but also increases variability. In contrast, experimental and image-based studies provide greater control over data acquisition but have more limited generalizability to routine farm conditions.

Critical analysis

Methodological heterogeneity in ultrasonographic studies is not a secondary issue but a major reason for variability in reported results. Some apparent differences in diagnostic performance may reflect differences in study design rather than true differences between diagnostic methods. Therefore, diagnostic accuracy should be interpreted in the context of the specific study conditions, including timing of examination, probe characteristics, reference standard, and unit of analysis. Direct pooling of numerical indicators without meta-analysis and without detailed stratification would therefore be methodologically inappropriate. Table 4 presents the main sources of methodological heterogeneity in ultrasonographic studies of early pregnancy diagnosis in sows.

#### 3.2.4. Sources of False Positive and False Negative Results

The main factor associated with false-negative results is the limited visualization of early gestational structures during the initial stages of gestation. This is a logical consequence of the biology of early gestation: before sufficient enlargement of the gestational vesicle and accumulation of fluid, visualization is uncertain. In A-mode, this corresponds to an insufficient acoustic signal, and in B-mode to an insufficiently clear morphological finding. The low negative predictive value reported by [5] during the early diagnostic window is consistent with this mechanism. False-positive results have different origins depending on the technology. In A-mode and pulse-mode, they may arise from interpretation of non-specific fluid structures. In B-mode, the main issue is biological rather than technical. If a true early pregnancy is detected ultrasonographically but subsequent embryonic mortality occurs and the animal does not farrow, the diagnosis will be classified as false positive when farrowing is used as the reference standard. This mechanism may contribute to the lower apparent specificity reported in studies using farrowing as the reference standard [4,18]. In algorithm-based image models, two types of errors should be distinguished. One corresponds to classical diagnostic errors analogous to clinical practice, while the other consists of computational errors related to annotation quality, image noise, or model transferability to a new dataset. This means that false-positive and false-negative results in AI models have both biological and technical dimensions.

Critical analysis

The systematic analysis of false-positive and false-negative results shows that ultrasonographic diagnosis in sows cannot be evaluated solely by numerical indicators without biological context. In A-mode, errors are predominantly methodological and related to indirect signal detection. In B-mode, a substantial proportion of false-positive results are biologically determined and do not necessarily reflect a true diagnostic error at the time of examination. This is particularly important for a high-quality systematic review, as it changes the interpretation of specificity. In AI-based approaches, errors should also be interpreted in terms of dataset quality, annotation reliability, and algorithmic generalization rather than only as clinical diagnostic inaccuracy. Table 5 presents the main sources of false-positive and false-negative results in ultrasonographic pregnancy diagnosis in sows.

The analysis of the available studies shows that the diagnostic performance of ultrasonographic methods depends simultaneously on the technology used, the gestational age at examination, and the clinical context of application. The differences between acoustic, imaging, and algorithm-based approaches are reflected both in diagnostic accuracy and in their practical applicability under conditions of routine reproductive diagnostics.

### 3.3. Progesterone-Based Methods for Early Pregnancy Diagnosis in Sows

#### 3.3.1. Diagnostic Principle and Biological Basis

Progesterone-based methods for early pregnancy diagnosis in sows are based on the physiology of the corpus luteum and the timing of luteolysis in non-pregnant animals. After ovulation, the corpora lutea secrete progesterone, which maintains the uterine environment necessary for early development of the conceptus. In non-pregnant sows, this secretory pattern is interrupted with the onset of luteolysis, whereas in pregnant animals progesterone secretion is maintained. The diagnostic principle is therefore indirect: the method does not directly demonstrate the presence of an embryo or gestational sac, but evaluates whether luteal function persists at a time when it should already have regressed in non-pregnant animals. This is the reason why progesterone is particularly useful for distinguishing non-pregnant from potentially pregnant animals after the expected period of luteolysis, but is less specific for confirming viable pregnancy [8,9]. The clinical value of this principle is directly related to the timing of testing. If sampling is performed too early, progesterone concentrations in pregnant and non-pregnant sows may be similar, as luteolysis has not yet been completed in non-pregnant animals. If performed within the appropriate window, low progesterone values have strong value for excluding pregnancy. This means that progesterone-based diagnostics function more effectively as a method for early identification of non-pregnant sows than as a method for definitive confirmation of viable pregnancy. This biological feature should determine the interpretation of results in individual publications [22].

Critical analysis

The most significant limitation of progesterone-based methods is not analytical but biological. Even with highly sensitive immunoassays, the measured variable remains a function of the corpus luteum and not a direct marker of the presence of a developing conceptus. Accordingly, a positive progesterone result should be interpreted as evidence of persistent luteal activity rather than as direct confirmation of a viable pregnancy. From this perspective, progesterone has a different diagnostic objective than ultrasonography: it identifies animals in which the luteal profile is compatible with pregnancy, but does not morphologically confirm pregnancy. This distinction should be considered in any direct comparison between hormonal and ultrasonographic methods.

#### 3.3.2. Blood-Based Progesterone Assays: Plasma, Serum and Blood Spots

The earliest and most established progesterone-based approaches in sows used blood samples. Claus [8] developed a microtiter plate enzyme immunoassay for determining progesterone in plasma and validated it by comparison with radioimmunoassay methods. Although complete diagnostic metrics for early pregnancy are not reported in the available record this study has methodological importance, as it demonstrates the transition from classical RIA to non-radioactive immunoassay systems more suitable for routine use. The next stage involves simplification of sampling. Lin and Hwang [9] used a blood paper method, while Moriyoshi et al. [21] and Chadio et al. [22] applied direct radioimmunoassay to blood collected on filter paper. Chadio et al. [22] clearly defined the study design: pregnancy was assessed in 95 sows on days 17–22 after artificial insemination, and the cut-off value was 7.5 ng/mL, calculated from concentrations in non-inseminated animals on the same days. A total of 85 animals were classified as pregnant according to the progesterone criterion, and 10 as non-pregnant. Positive accuracy was 98.8%, negative accuracy was 80%, and overall accuracy was 96.8%. These results indicate that, with an appropriately selected time window and threshold, progesterone analysis in blood spots on filter paper can be a useful tool for early pregnancy screening [22].

The main practical advantage of the dried blood spot approach is logistical. It allows collection of a small volume of blood, drying on a carrier, easy transport, and subsequent laboratory analysis without immediate processing of the sample. This is particularly important in production systems where access to a laboratory is not immediate. At the same time, the method remains dependent on sampling quality, uniform application on the paper, storage conditions, and extraction efficiency. Therefore, the method improves practical applicability, but it also introduces new pre-analytical sources of variability [9,21].

Critical analysis

The comparison between plasma/serum and dried blood spot approaches shows a classical trade-off between analytical control and field applicability. Blood laboratory samples provide a more direct and stable matrix but require venipuncture, processing, and transport. Blood on filter paper is more practical but introduces pre-analytical variability. Nevertheless, the data reported by Chadio et al. [22] show that, with a clearly defined cut-off and appropriate timing of testing, diagnostic performance can remain high. Methodologically, it is important that even with a high overall accuracy of 96.8%, negative accuracy remained 80%, indicating that some animals classified as non-pregnant subsequently farrowed. This indicates that, despite high overall accuracy, interpretation of negative results remains sensitive to the selected threshold and to biological variability around luteolysis.

#### 3.3.3. Timing of Sampling and Its Effect on Diagnostic Interpretation

The timing of sampling is the main factor determining the diagnostic value of progesterone tests. In blood-based assays, the window of highest usefulness begins after the expected luteolysis. This is demonstrated in the study by Chadio et al. [22], where sampling was performed between days 17 and 22, and the cut-off was calculated based on non-pregnant animals within the same time interval. This is an important methodological detail, as it shows that the threshold is not universal, but depends on the biological stage at which the population is evaluated. The same issue is evident in later or alternative matrices. Moriyoshi et al. [10] investigate progesterone in saliva between days 17 and 24, and [11] measure fecal gestagens between days 21 and 25. There is an important difference in the logic of these studies: blood reflects circulating progesterone more directly and earlier, whereas saliva and especially feces involve additional biological processes of diffusion, metabolism, and excretion. Therefore, it is not surprising that the time window in fecal tests is shifted toward later days. The field ELISA study of [12] is also important in this context, as it positions the serum progesterone test around day 17 after insemination. The cut-off for positivity is >5 ng/mL, and high sensitivity and specificity are reported. The difference between the 7.5 ng/mL threshold reported by Chadio et al. [22] and the >5 ng/mL threshold reported in the field ELISA study [12] illustrates that progesterone cut-off values were not standardized across studies. These thresholds should therefore be interpreted in relation to the sampling day, biological matrix, analytical method, and population used for threshold definition. This places day 17 as a practical compromise point: sufficiently early for management decisions, but also sufficiently late to more clearly distinguish between pregnant and non-pregnant animals.

Critical analysis

The differences between days 17–22, 17–24, and 21–25 are not merely formal. They reflect different biological and analytical strategies. The earlier the test is performed, the greater the risk of overlap between the profiles of pregnant and non-pregnant animals. The later it is performed, the higher the probability of reliable differentiation, but the advantage of very early diagnosis is reduced. This means that neither the optimal day of testing nor the diagnostic cut-off can be considered universal for all progesterone-based methods. Both must be interpreted in the context of the biological matrix, analytical method, sampling day, and practical objective of the test. Table 6 presents timing, sample matrix, and analytical characteristics of progesterone-based studies.

#### 3.3.4. Saliva and Feces as Alternative Biological Matrices

Saliva and feces have been proposed as less invasive or completely non-invasive alternatives to blood samples for progesterone determination in sows. Moriyoshi et al. [10] investigated the possibility of measuring progesterone in saliva using an enzyme immunoassay based on a bovine milk progesterone qualitative test EIA kit. Saliva samples were collected by placing an absorbent swab in the oral cavity of the animal for a short period, after which the collected saliva is extracted and analyzed using the immunoassay system. In this study, samples were collected between day 17 and day 24 after insemination, corresponding to the period after the expected luteolysis in non-pregnant animals [10]. An alternative non-invasive approach is the determination of gestagen metabolites in feces. Moriyoshi et al. [11] used an enzyme immunoassay for quantitative determination of progesterone metabolites in fecal samples. Fecal samples were collected immediately after defecation to avoid degradation of metabolites, after which they are subjected to extraction prior to analysis. In this study, sampling was performed between days 21 and day 25 after insemination, with the later diagnostic window reflecting the time delay between circulating progesterone and the appearance of its metabolites in feces [11]. Saliva has the advantage of containing the free fraction of steroid hormones, which diffuses from the blood through the salivary glands and can therefore reflect circulating progesterone concentrations. Collection of saliva is relatively easy and does not require specialized equipment or venipuncture, which reduces stress in animals and facilitates sampling under production conditions. Fecal samples, on the other hand, represent a completely non-invasive matrix and can be collected without direct contact with the animal, making them practically applicable with minimal handling.

Despite these advantages, both matrices are characterized by important biological and analytical limitations. The concentration of progesterone in saliva is significantly lower than in blood and may be influenced by the rate of salivary secretion, local physiology of the oral cavity, and the degree of sample contamination. In fecal tests, the measured compounds represent progesterone metabolites formed after hepatic metabolism and excreted via bile. This means that their concentration in feces reflects an integrated hormonal signal over a period of time and does not directly correspond to the instantaneous concentrations of circulating progesterone.

Critical analysis

The comparison between blood, saliva, and fecal samples shows a substantial difference in the biological signal being measured. Blood-based tests determine circulating progesterone directly and therefore allow earlier diagnosis after the onset of luteolysis. Saliva reflects the free fraction of the hormone but at significantly lower concentrations, which may reduce analytical sensitivity. Fecal tests measure progesterone metabolites, which introduces an additional time delay between hormonal secretion and the measured signal. These differences mean that the diagnostic window and interpretation of results differ between matrices and cannot be directly transferred from blood-based assays to saliva- or feces-based methods. While blood-based tests can be interpreted around day 17–22, saliva-based tests are used approximately between day 17–24, and fecal analyses between day 21–25 [10,11]. Therefore, non-invasive samples can expand diagnostic possibilities, but their interpretation requires careful consideration of timing, biological matrix, and the fact that progesterone-based methods reflect luteal activity rather than direct embryo viability.

Methodological differences between studies are not limited to the analytical method used, but also include differences in the biological matrix and the sampling procedure. These factors influence the diagnostic window, analytical sensitivity, and interpretation of results. Table 7 provides a matrix-level comparison of progesterone-based approaches and summarizes how the biological sample influences the diagnostic window, analytical sensitivity, practical applicability, and interpretation of results.

#### 3.3.5. Diagnostic Errors and Practical Interpretability

In progesterone-based methods, false-positive and false-negative results arise from the diagnostic principle itself. A false-positive result is usually not an analytical error but a biological mismatch between luteal function and the actual status of embryonic development. If corpora lutea persist in the absence of a viable pregnancy, as may occur in pseudopregnancy, progesterone concentrations may remain high and the animal may be classified as pregnant. A false-negative result most commonly occurs due to inappropriately early sampling or an incorrectly selected threshold around the critical transition period of luteolysis. This is demonstrated by [22], where two of the ten sows classified as non-pregnant subsequently farrowed, explaining a negative accuracy of 80% despite a high overall accuracy.

These diagnostic errors have important practical implications. Progesterone is more suitable for screening-out non-pregnant animals than for definitive confirmation of pregnancy. This does not make it a weak method. In the context of reproductive management, early identification of non-pregnant sows is highly valuable. In the case of a positive result, the progesterone test should be interpreted as an indicator of persistent luteal function rather than as direct evidence of a viable conceptus.

Critical analysis

The strength of progesterone-based methods should not be evaluated using the model of ultrasonographic diagnosis. If direct confirmation of pregnancy is required, progesterone-based methods are conceptually more limited than ultrasonography. However, if the objective is early differentiation of sows that have not maintained luteal function after insemination, the method has high practical value. On this basis, progesterone-based diagnostics should be interpreted as a tool for reproductive management rather than as a morphological test for confirmation of pregnancy. This is the reason why it has a different, rather than inferior, role compared to ultrasonography in early pregnancy diagnosis in sows.

The main mechanisms leading to false-positive and false-negative results in progesterone-based tests are summarized in Table 8.

Diagnostic errors in progesterone-based methods arise mainly from the biological basis of the test and from the timing of sampling. This explains the high value of these methods for early identification of non-pregnant animals, but also limits their ability to directly confirm viable pregnancy.

### 3.4. Alternative Endocrine and Biomarker Approaches

In addition to ultrasonographic and progesterone-based methods, several studies have evaluated alternative endocrine or biomarker-based approaches for early pregnancy diagnosis in sows, including estrone sulfate or estrone-based measurements, early pregnancy factor detection, and metabolomic profiling.

Estrone sulfate has been investigated in plasma, serum, urine, and fecal samples as an endocrine marker associated with conceptus development and pregnancy-related endocrine activity [13,14,23,24,25,26,27,28]. Early pregnancy factor detection has also been proposed as a potential approach for early pregnancy diagnosis [29], and salivary metabolomic profiling has more recently been explored as a non-invasive method for identifying pregnancy-associated metabolic patterns [30].

The available evidence for these alternative biomarkers remains less extensive and less standardized than that for ultrasonography and progesterone-based assays. Their diagnostic usefulness depends on timing of sampling, biological matrix, analytical method, and validation under field conditions. These approaches should currently be interpreted as complementary or exploratory rather than as established routine methods for early pregnancy diagnosis in sows.

### 3.5. Comparative Evaluation of Ultrasonography and Progesterone-Based Methods

The comparative analysis of the included studies shows that ultrasonographic and progesterone-based methods have different diagnostic functions in the early assessment of pregnancy in sows. Ultrasonographic techniques provide morphological information through evaluation of the uterus and gestational structures, whereas progesterone tests provide functional information on luteal activity [3,5,8,9]. Therefore, the two approaches are not interchangeable, but address different diagnostic tasks. The main practical difference between them is related to the nature of the diagnostic conclusion. Ultrasonographic methods allow direct confirmation of pregnancy when gestational structures are sufficiently developed for reliable visualization [3,5]. Progesterone-based tests are more suitable for early identification of non-pregnant animals after the expected luteolysis, but a positive result is not equivalent to morphologically confirmed pregnancy [9,10,11,12]. This means that ultrasonography has higher value for confirming pregnancy, whereas progesterone-based tests have higher value for early exclusion of non-pregnancy. The differences between the two approaches are also reflected in the sources of diagnostic error. In ultrasonographic methods, accuracy depends mainly on gestational age, the equipment used, and the quality of interpretation [4,5]. In progesterone-based tests, the main limitations are related to timing of sampling, threshold value, and the biological mismatch between persistent luteal function and the actual presence of a viable pregnancy [8,9,10,11,12].

The practical value of both approaches is most evident when they are considered as complementary methods in reproductive management. Progesterone-based tests can be used for early selection of sows in which pregnancy is unlikely, whereas ultrasonography can be used for subsequent confirmation of pregnancy through direct evaluation of the uterus [3,5,9,12]. From a farm-adoption perspective, the two methods also differ in equipment requirements, time per diagnosis, operator training, and cost structure. Ultrasonography requires a scanner and a trained operator but provides an immediate animal-level result [3,5]. Progesterone-based assays may be easier to standardize analytically, but they require sample collection, handling, and laboratory or kit-based analysis, and the result reflects luteal function rather than direct pregnancy visualization [3,4,5,8,9,10,11,12]. Table 9 presents a summarized comparison between the two diagnostic approaches.

Table 9 shows that the differences between the two methods are not only technical but also arise from the biological basis of each diagnostic approach. Ultrasonography is aimed at visual confirmation of pregnancy, whereas progesterone-based tests assess whether the endocrine profile is compatible with pregnancy [3,5,8,9]. This determines their role in practice: ultrasonographic methods have higher value for confirming pregnancy, whereas progesterone-based approaches are more suitable for early identification of non-pregnant animals [3,5,9,12].

## 4. Perspectives and Future Directions

The analysis of the available studies shows that early pregnancy diagnosis in sows remains an area with pronounced methodological differences between individual studies. A substantial proportion of publications use different diagnostic time windows, different biological samples, and different reference standards for confirmation of pregnancy. These differences make direct comparison of diagnostic accuracy between methods difficult.

In ultrasonographic methods, future development is mainly related to standardization of diagnostic protocols and improvement of ultrasonographic image interpretation. Recent studies show increasing interest in the use of algorithms for analysis of ultrasound images and automated detection of gestational structures [6,7,19,20]. These approaches may reduce the dependence of diagnosis on operator experience and improve standardization of interpretation. However, their future implementation requires validation under routine farm conditions and evaluation of whether their cost, technical requirements, and training demands are justified compared with examination by an experienced operator using conventional portable ultrasonography.

In progesterone-based methods, the main direction of development is related to optimization of the diagnostic window and improvement of the analytical sensitivity of the tests. Studies on alternative biological matrices, such as saliva and feces, indicate the potential for development of non-invasive diagnostic approaches that can be applied with minimal handling of animals [10,11].

The available literature also indicates the need for better standardization of diagnostic criteria in future studies. Differences in progesterone threshold values, timing of sampling, and reference standards used can significantly influence the reported diagnostic accuracy. More unified protocols, including standardized timing of examination, clearly defined reference standards, consistent reporting of sensitivity and specificity, and transparent description of cut-off values, would allow more reliable comparison between diagnostic approaches and may enable future quantitative synthesis or meta-analysis.

## 5. Limitations of the Review

The present systematic review includes studies with different methodological characteristics, which limits direct quantitative comparison between the individual diagnostic methods. The publications differ with respect to the timing of diagnostic examination after insemination, the ultrasonographic techniques used, the type of transducers, and the analytical methods for progesterone determination. There are also differences in the biological samples used for progesterone analysis, including plasma, serum, blood spots on filter paper, saliva, and feces. These differences may affect the measured hormone concentrations and the interpretation of diagnostic results. An additional limitation is the variation in reference criteria for confirmation of pregnancy. In some studies, the final reproductive status is determined by subsequent farrowing, while in others repeated ultrasonographic diagnosis or clinical observation is used. These differences may influence the evaluation of diagnostic accuracy of the individual methods. In addition, several studies did not report complete diagnostic performance indicators, such as sensitivity, specificity, or accuracy, which limited direct comparison of diagnostic performance across all included publications. Another limitation is that studies evaluating artificial intelligence or machine-learning approaches were mainly based on image datasets or experimental validation, and their results may not be directly generalizable to routine field conditions. Due to substantial differences in study design and diagnostic protocols, a meta-analysis was not performed. The present review therefore used a structured narrative synthesis rather than quantitative pooling of diagnostic accuracy estimates.

## 6. Conclusions

The available literature shows that early pregnancy diagnosis in sows can be performed using two main diagnostic approaches: ultrasonographic diagnosis and progesterone-based testing. These methods provide different types of diagnostic information and should therefore be interpreted as complementary rather than interchangeable approaches. Progesterone-based tests provide functional information on luteal activity and are most useful for the early identification of non-pregnant animals after the expected period of luteolysis. However, a positive progesterone result should not be interpreted as definitive confirmation of viable pregnancy, because elevated progesterone reflects persistent luteal function rather than direct visualization of a conceptus.

Ultrasonographic methods provide morphological information and allow direct evaluation of uterine and gestational structures. Their diagnostic value increases with appropriate timing of examination, adequate image quality, and operator experience. Real-time B-mode ultrasonography remains the most practical method for confirming pregnancy under farm conditions, whereas AI-assisted image analysis represents a promising but still insufficiently field-validated development. From a practical perspective, the most appropriate diagnostic strategy depends on the purpose of testing: progesterone-based assays are suitable for early reproductive screening, particularly for identifying animals unlikely to be pregnant, while ultrasonography is more suitable for confirmation of pregnancy. The combined and sequential use of these approaches may improve reproductive decision-making in sow herds by linking early screening with subsequent morphological confirmation.

## Figures and Tables

**Table 1:** PRISMA flow of study selection for the systematic review.

Stage	Description	Number of Records
Identification	Records identified through database searching (Web of Science, Scopus, PubMed)	186
	Additional records identified through citation searching	12
Screening	Records after duplicates removed	148
	Records screened (titles and abstracts)	148
	Records excluded (irrelevant topic, non-swine species, not related to pregnancy diagnosis)	96
Eligibility	Full-text articles assessed for eligibility	52
	Full-text articles excluded with reasons (review articles, insufficient methodological data, not focused on early pregnancy diagnosis)	22
Included	Studies included in qualitative synthesis	30

**Table 2:** Characteristics of included studies investigating early pregnancy diagnosis in sows.

Study	Method	Sample	Timing after Insemination	Reference Standard	Reported Diagnostic Metrics
[1]	A-mode ultrasound	abdominal scan	early gestation	pregnancy outcome	NR
[15]	Pulse-mode ultrasound	abdominal scan	early gestation	pregnancy outcome	NR
[2]	Linear electronic ultrasound	abdominal scan	early gestation	pregnancy outcome	NR
[3]	Real-time ultrasound vs. A-mode	abdominal scan	early gestation	pregnancy outcome	NR
[16]	A-mode ultrasound vs. palpation	abdominal scan	early gestation	pregnancy outcome	Accuracy 96.8%, Sensitivity 98.9%
[17]	Ultrasound evaluation of embryo development	ultrasound	15–25 d	pregnancy outcome	NR
[5]	Trans-abdominal ultrasound	ultrasound	16–25 d	pregnancy outcome	Accuracy >95% after day 23–24
[4]	Real-time ultrasound	ultrasound	26–30 d	farrowing	Sensitivity 99%, Specificity 73.1%
[18]	Real-time ultrasound	ultrasound	<30 d	farrowing	Sensitivity 84.21%, Specificity 75%
[6]	Machine learning ultrasound analysis	ultrasound images	mid-gestation	litter size	RMSE 0.91–1.05
[19]	AI-based ultrasound diagnosis	ultrasound images	early gestation	pregnancy status	Sensitivity 0.9876, Specificity 0.9982
[7]	YOLOv7 ultrasound detection	ultrasound images	early gestation	image annotation	mAP 89.8%
[20]	Deep learning ultrasound	ultrasound images	early gestation	pregnancy status	AUC ≈0.86
[8]	Progesterone enzyme immunoassay	plasma	early gestation	RIA comparison	NR
[9]	Progesterone assay (blood paper)	blood	early gestation	pregnancy outcome	NR
[21]	Direct RIA progesterone	blood	early gestation	pregnancy outcome	NR
[10]	Progesterone EIA	saliva	17–24 d	pregnancy outcome	NR
[11]	Gestagen EIA	feces	21–25 d	pregnancy outcome	NR
[12]	Progesterone ELISA	serum	~17 d	pregnancy outcome	NR
[22]	Direct RIA progesterone	blood	early gestation	pregnancy outcome	Positive accuracy 98.8%, overall accuracy 96.8%
[13]	Estrone sulfate detection	plasma	~17 d	pregnancy outcome	detection ~50% of pregnant sows
[23]	Estrone sulfate assay	serum	early gestation	pregnancy outcome	NR
[24]	Estrone sulfate EIA	serum	early gestation	pregnancy outcome	NR
[14]	Estrone sulfate vs. Doppler US	plasma	24–30 d	pregnancy outcome	Sensitivity >94%, Specificity 78%
[25]	Estrone sulfate EIA	plasma	25–30 d	pregnancy outcome	NR
[26]	Estrone ELISA	feces	early gestation	ultrasound comparison	NR
[27]	Estrone sulfate measurement	plasma	mid gestation	litter size	NR
[28]	Estrone sulfate measurement	serum/urine	mid gestation	litter size	NR
[29]	Early pregnancy factor antibody	urine	~20 d	pregnancy status	NR
[30]	Salivary metabolomics	saliva	early gestation	pregnancy status	AUC 0.823–0.938

NR—not reported.

**Table 3:** Reported diagnostic performance of ultrasonographic approaches in sows.

Ultrasonographic Approach	Diagnostic Target	Timing After Insemination	Reported Metric(s)	Interpretation
A-mode ultrasound	indirect detection of intrauterine fluid	early gestation	Accuracy 96.8%; Sensitivity 98.9%	high sensitivity, limited structural specificity
Real-time B-mode ultrasound	visualization of gestational structures	18–30 days	>95% Se/Sp/accuracy after day 23–24; Se 99%; Sp 73.1%; Se 84.21%; Sp 75%	reliable after appropriate gestational age; performance varies by study design
AI-based ultrasound analysis	classification or detection on ultrasound images	early gestation	Se 0.9876; Sp 0.9982; mAP 89.8%; AUC ~0.86	high internal performance; limited field-level validation

**Table 4:** Methodological heterogeneity in ultrasonographic studies of early prenancy diagnosis in sows.

Source of Heterogeneity	Variation Across Studies	Expected Impact on Diagnostic Performance
Day of examination	15–30 days after insemination	Earlier diagnosis increases the risk of false-negative results; later diagnosis may increase apparent sensitivity
Probe type and frequency	Linear versus sector probes; 5 MHz versus 3.5 MHz	Affects image resolution and penetration depth
Reference standard	Pregnancy status, farrowing outcome, or image-based validation	Influences apparent specificity and clinical interpretation
Study setting	Experimental versus field conditions	Field studies increase variability but improve external validity
Unit of analysis	Animal-level diagnosis versus image-level model performance	Limits direct comparison between conventional ultrasound and AI-based studies

**Table 5:** Main sources of false -positive and false-negative results in ultrasonographic pregnancy diagnosis in sows.

Method	Main Source of False Positives	Main Source of False Negatives	Dominant Limitation
A-mode ultrasound	Nonspecific fluid-related echoes	Insufficient intrauterine fluid in early gestation	Indirect diagnostic signal
Pulse-mode ultrasound	Nonspecific echo interpretation	Weak early signal	No structural visualization
Real-time B-mode ultrasound	Embryonic loss after diagnosis; interpretation near threshold	Very early examination	Dependence on gestational age and operator skill
AI/image-based analysis	Annotation bias; dataset shift; image artefacts	Low-quality images; poor generalization	Limited external validation

**Table 6:** Timing, sample matrix and analytical characteristics of progesterone-based studies in sows.

Study	Matrix	Sampling Method	Day After Insemination	Analytical Method	Cut-Off/Reported Threshold	Reported Diagnostic Data
[8]	Plasma	venous blood sampling	early gestation	microtitreplate EIA vs. RIA	NR	analytical validation; diagnostic metrics NR
[9]	Blood on filter paper	blood spot on paper	early gestation	progesterone assay	NR	NR
[21]	Blood on filter paper	dried blood spot	early gestation	direct RIA	NR	NR
[22]	Blood on filter paper	dried blood spot	17–22	direct RIA	7.5 ng/mL	positive accuracy 98.8%; negative accuracy 80%; overall accuracy 96.8%
[10]	Saliva	saliva collection	17–24	EIA	NR	diagnostic approach reported; detailed metrics NR in accessible text
[11]	Feces	fecal collection	21–25	EIA for gestagens	NR	diagnostic approach reported; detailed metrics NR in accessible text
[12]	Serum	blood sampling	~17	ELISA	>5 ng/mL positive	high sensitivity and specificity reported; complete diagnostic metrics not available in the accessible text

**Table 7:** Comparative analytical profile of progesterone-based methods in sows.

Approach	What is Measured	Typical Diagnostic Window (Days Post-Insemination)	Practical Advantage	Main Biological Limitation	Main Analytical/Methodological Limitation	Most Suitable Use
Plasma/serum progesterone	circulating progesterone	~17–22	direct hormonal assessment	reflects luteal function, not embryo viability	requires venipuncture and laboratory processing	early identification of non-pregnant sows
Blood on filter paper	circulating progesterone extracted from dried blood spot	~17–22	easier transport and storage of samples	same biological limitation as blood assays	variability in blood volume and extraction efficiency	field-adapted hormonal screening
Salivary progesterone	free fraction of progesterone diffusing into saliva	~17–24	non-invasive sampling	lower hormone concentrations than plasma	possible contamination and sampling variability	supportive non-invasive testing
Fecal gestagens	progesterone metabolites excreted in feces	~21–25	completely non-invasive sampling	delayed reflection of endocrine status	metabolite-based interpretation and time lag	delayed non-invasive screening

**Table 8:** Sources of false-positive and false-negative results in progesterone-based pregnancy diagnosis in sows.

Type of Diagnostic Error	Biological Mechanism	Diagnostic Consequence	Methodological Context
False positive result	persistent corpora lutea or pseudopregnancy without viable pregnancy	progesterone concentrations remain elevated despite absence of viable embryos	hormonal tests based solely on luteal function
False positive result	early embryonic mortality after maternal recognition of pregnancy	progesterone remains elevated although pregnancy does not continue	testing performed after embryonic loss
False negative result	sampling before completion of luteolysis in non-pregnant animals	progesterone concentrations still elevated in non-pregnant sows	early sampling before ~17 days post insemination
False negative result	inappropriate diagnostic threshold	misclassification due to overlap between progesterone levels in pregnant and non-pregnant animals	poorly defined cut-off values
False negative result	analytical variability in sample matrix	inaccurate hormone measurement	sample degradation or extraction variability

**Table 9:** Comparative characteristics of ultrasonographic and progesterone-based approaches for early pregnancy diagnosis in sows.

Diagnostic Approach	Diagnostic Principle	Main Diagnostic Strength	Main Limitation	Practical Role in Reproductive Management	Practical Constraints
Ultrasonographic	Direct visualization of uterine and embryonic structures	Morphological confirmation of pregnancy	Operator dependence; equipment requirement	Confirmation of pregnancy	Requires ultrasound equipment and trained operator; result available immediately
Progesterone-based assays	Evaluation of luteal endocrine activity	Early identification of non-pregnant animals	Cannot confirm presence of viable embryos	Early reproductive screening	Requires sample collection and assay processing; positive results require careful interpretation

## Data Availability

No new data were created or analyzed in this study.

## Supplementary Materials

The following supporting information can be downloaded at: https://www.mdpi.com/article/10.3390/life16050854/s1, Supplementary Table S1.

## References

[1] Lindahl I.L., Murison P.J., Shank R.L. (1975). Early diagnosis of pregnancy in sows by ultrasonic amplitude-depth analysis. J. Anim. Sci..

[2] Inaba T., Nakazima N., Matsui N., Imori T. (1983). Early pregnancy diagnosis in sows by ultrasonic linear electronic scanning. Theriogenology.

[3] Taverne M.A.M., Oving L., van Lieshout M., Willemse A.H. (1985). Pregnancy diagnosis in pigs: A field study comparing linear-array real-time ultrasound scanning and amplitude-depth analysis. Vet. Q..

[4] Szenci O., Palme R., Taverne M.A.M., Varga J., Meersma N., Wissink E. (1997). Evaluation of false ultrasonographic pregnancy diagnoses in sows. Theriogenology.

[5] Maes D., Dewulf J., Vanderhaeghe C., Claerebout K., de Kruif A. (2006). Accuracy of trans-abdominal ultrasound pregnancy diagnosis in sows using a linear or sector probe. Reprod. Domest. Anim..

[6] Kousenidis K., Kirtsanis G., Karageorgiou E., Tsiokos D. (2022). Evaluation of a numerical, real-time ultrasound imaging model for the prediction of litter size in pregnant sows, with machine learning. Animals.

[7] Kim T.-k., Kim J.S., Cho H.-C. (2023). Deep-learning-based gestational sac detection in ultrasound images using modified YOLOv7-E6E model. J. Anim. Sci. Technol..

[8] Claus V.R. (1985). Development of a microtitreplate enzyme immunoassay for progesterone determination in pig blood plasma and its validation and comparison to radioimmunological methods. Reprod. Domest. Anim..

[9] Lin J.H., Hwang B. (1988). Early pregnancy diagnosis in sows by progesterone assay with blood paper method. Br. Vet. J..

[10] Moriyoshi M., Tamaki M., Nakao T., Kawata K. (1996). Early pregnancy diagnosis in the sow by saliva progesterone measurement using a bovine milk progesterone qualitative test EIA kit. J. Vet. Med. Sci..

[11] Moriyoshi M., Nozoki K., Ohtaki T., Nakada K., Nakao T., Kawata K. (1997). Measurement of gestagen concentration in feces using a bovine milk progesterone quantitative test EIA kit and its application to early pregnancy diagnosis in the sow. J. Vet. Med. Sci..

[12] Boma M.H., Bilkei G. (2008). Field experiences with early pregnancy diagnosis by progesterone-based ELISA in sows. Onderstepoort J. Vet. Res..

[13] Robertson H.A., King G.J., Dyck G.W. (1978). The appearance of oestrone sulphate in the peripheral plasma of the pig early in pregnancy. J. Reprod. Fertil..

[14] Atkinson S., Buddle J.R., Williamson P., Hawkins C.D., Wilson R.H. (1986). A comparison between plasma oestrone sulphate concentration and Doppler ultrasound as methods for pregnancy diagnosis in sows. Theriogenology.

[15] Holtz W. (1982). Pregnancy detection in swine by pulse-mode ultrasound. Anim. Reprod. Sci..

[16] Pyörälä S. (1989). Pregnancy diagnosis in swine by palpation and by amplitude-depth ultrasound scanning. Theriogenology.

[17] De Rensis F., Bigliardi E., Parmigiani E., Peters A.R. (2000). Early diagnosis of pregnancy in sows by ultrasound evaluation of embryo development and uterine echotexture. Vet. Rec..

[18] Gokuldas P.P., Shinde K., Naik S., Chakurkar E.B. (2023). Assessment of diagnostic accuracy and effectiveness of transabdominal real-time ultrasound imaging for pregnancy diagnosis in breeding sows under intensive management. Trop. Anim. Health Prod..

[19] Chae J.-W., Choi Y.-H., Lee J.-N., Park H.-J., Jeong Y.-D., Cho E.-S., Kim Y.-S., Kim T.-K., Sa S.-J., Cho H.-C. (2023). An intelligent method for pregnancy diagnosis in breeding sows according to ultrasonography algorithms. J. Anim. Sci. Technol..

[20] Kim T.-k., Choi Y.-H., Hong J.-S., Park H.-J., Kim Y.-M., Kim J.-E., Lee J.-H., Sa S.-J., Jeong Y.-D., Kim J.-S. (2025). Deep learning-enhanced diagnosis of sow pregnancy through low-frequency ultrasound imaging. Animals.

[21] Moriyoshi M., Tamaki M., Nakao T., Kawata K. (1994). Early pregnancy diagnosis in swine by direct radioimmunoassay for progesterone in blood spotted on filter paper. J. Reprod. Dev..

[22] Chadio S., Xylouri E., Kalogiannis D., Michalopoulou E., Evagelatos S., Menegatos I. (2002). Early pregnancy diagnosis in swine by direct radioimmunoassay for progesterone in blood spotted on filter paper. Anim. Reprod. Sci..

[23] Hattersley J.P., Robertson H.A., Dyck G.W. (1980). Estimation of oestrone sulphate in the serum of pregnant sows. J. Reprod. Fertil..

[24] Sugiyama S., Nakao T., Tsunoda N., Kawata K. (1985). An enzymeimmunoassay of serum oestrone sulphate and its application to early pregnancy diagnosis in pigs. Br. Vet. J..

[25] van de Wiel D.F.M., Vos E.A., te Brake J.H.A., van der Lende T. (1992). Pregnancy diagnosis in sows: Enzyme immunoassay measurement of oestrone sulphate in plasma on days 25–30 after insemination in comparison to ultrasound scanning on day 28. Livest. Prod. Sci..

[26] Vos E.A., van Oord R., Taverne M.A.M., Kruip T.A.M. (1999). Pregnancy diagnosis in sows: Direct ELISA for estrone in feces and its prospects for an on-farm test, in comparison to ultrasonography. Theriogenology.

[27] Stone R.T., Maurer R.R., Reddy V.B. (1986). Oestrone sulphate levels in plasma of sows as a basis for prediction of litter size at term. Anim. Reprod. Sci..

[28] Frank G.R., Maurer R.R., Reddy V.B. (1987). Direct estimation of estrone sulfate in serum and urine of pregnant swine as indicators of litter size at birth. Anim. Reprod. Sci..

[29] Park S., Cho E.-S., Kim C.-H., Lee S., Jeong Y.-D., Park M., Kim D., Seo D., Kim Y.-H., Hochi S. (2024). Detection of sow pregnancy in day-20 urine samples using monoclonal antibody against synthesized porcine early pregnancy factor: Preliminary results. Theriogenology.

[30] Ren Y., Zhang Q., He F., Qi M., Fu B., Zhang H., Huang T. (2024). Metabolomics reveals early pregnancy biomarkers in sows: A non-invasive diagnostic approach. Front. Vet. Sci..

