# Supplementary material for: Early Pregnancy Diagnosis in Sows: A Comparative Evaluation of Ultrasonographic and Progesterone-Based Methods
Source: Life (Basel). 2026 May 21;16(5):854. doi: 10.3390/life16050854 (PMC13208898; doi:10.3390/life16050854)
Supplement: Supplementary file 1 [file life-16-00854-s001.zip › life-4277516-supplementary.pdf]

Supplementary Table S1. Quality assessment of included studies using a simplified QUADAS-2 framework

Study	Patient Selection	Index Test	Reference Standard	Flow and Timing
Lindahl et al. (1975)	Unclear	Low	Unclear	Unclear
Inaba et al. (1983)	Unclear	Low	Unclear	Unclear
Taverne et al. (1985)	Low	Low	Low	Low
Szenci et al. (1997)	Unclear	Low	High	Unclear
Maes et al. (2006)	Low	Low	Low	Low
Holtz (1982)	Unclear	Low	Unclear	Unclear
Pyörälä (1989)	Unclear	Low	Low	Unclear
De Rensis et al. (2000)	Low	Low	Low	Low
Gokuldas et al. (2023)	Low	Low	Low	Low
Lin and Hwang (1988)	Low	Low	Low	Unclear
Moriyoshi et al. (1994)	Unclear	Low	Low	Unclear
Moriyoshi et al. (1996)	Unclear	Low	Low	Unclear
Moriyoshi et al. (1997)	Unclear	Low	Low	Unclear
Chadio et al. (2002)	Low	Low	Low	Low
Boma and Bilkei (2008)	Low	Low	Low	Low
Atkinson et al. (1986)	Low	Low	Low	Unclear
van de Wiel et al. (1992)	Low	Low	Low	Low
Vos et al. (1999)	Low	Low	Low	Unclear

Legend: Low = low risk of bias; High = high risk of bias; Unclear = insufficient information to assess risk of bias.
